# Perspective of using *in vitro* models to understand immunotherapy-induced cytokine release syndrome

**DOI:** 10.3389/fimmu.2025.1732193

**Published:** 2026-01-09

**Authors:** Ethan Perkins, Christopher Cooper, Emma Lund, Miriam Alb, Hannah Morgan, Birgit Fogal, Philip Hewitt, Alexander Mazein, Marek Ostaszewski, Katherina Sewald

**Affiliations:** 1 Institute of Cancer Therapeutics, University of Bradford, Bradford, United Kingdom; 2 Immunology & Immunotoxicology, Labcorp Early Development Laboratories Limited, Harrogate, United Kingdom; 3 Universitätsklinikum Würzburg, Medizinische Klinik und Poliklinik II, Lehrstuhl für Zelluläre Immuntherapie, Würzburg, Germany; 4 Novartis Pharma Aktiengesellschaft (AG), Biomedical Research, Novartis Campus, Basel, Switzerland; 5 Boehringer Ingelheim Pharmaceuticals Inc., Nonclinical Drug Safety, Ridgefield, CT, United States; 6 Merck HealthCare Kommanditgesellschaft auf Aktien (KGaA), Darmstadt, Germany; 7 Luxembourg Centre for Systems Biomedicine, University of Luxembourg, Belvaux, Luxembourg; 8 Fraunhofer Institute for Toxicology and Experimental Medicine, Hannover, Germany

**Keywords:** adverse outcome pathway (AOP), clinical relevance, immunotoxicology, microphysiological system, organ on chip, cytokine release syndrome

## Abstract

Since the TGN1412 clinical trial failure to predict cytokine release syndrome (CRS) during preclinical trials, alternative *in vitro* models have become increasingly important for identifying potential adverse outcomes in early drug development. Considering this, in 2019 the IMI2/EU immune safety avatar (imSAVAR) consortium was established, encompassing academic, industry, and regulatory organizations. ImSAVAR aims to deliver a broad range of tools to enhance our ability to assess the efficacy and safety of immunomodulatory therapies. In addition, imSAVAR uses the adverse outcome pathway (AOP) concept to describe immune-related adverse effects, such as CRS, thereby facilitating the discovery of new biological markers for clinical management and prediction of immune-related adverse effects in nonclinical development. ImSAVAR unanimously agreed that CRS and advanced cytokine release assay (CRA) development is a key focus with regards to immunological safety testing and hazard identification. The CRA field has rapidly accelerated in recent years, with the rise of New Approach Methodologies (NAMs) to provide enhanced predictive immunological safety testing within a clinical setting. Here, we provide an up-to-date review of recent developments of advanced, *in vitro* CRA models, discuss how these advances may impact the future field of nonclinical toxicology and the understanding of immune-related adverse outcomes and offer guidance on appropriate model selection.

## Introduction

Cytokine release syndrome (CRS) is an acute, systemic inflammatory response characterized by fever, hypotension, and organ dysfunction. It can arise in response to therapeutics or infections (1, 2). Several high-profile incidences of CRS have been reported in response to immunotherapies, most notable during the 2006 clinical trial of the CD28 superagonist TGN1412 monoclonal antibody (mAb) (3). The risk of initiating such immune-related adverse outcomes is an important consideration when developing therapeutics that interact with the immune system, as is the case for most large molecule drugs. In the past decade, several new T cell-engaging immunotherapies have been approved by regulatory bodies (4–6). In 2012, a young girl with acute lymphoblastic B-cell leukemia relapsed after her body resisted chemotherapy for 16 months, leading to a terminal diagnosis. She was enrolled in a clinical trial for CD19 chimeric antigen receptor (CAR) T cell immunotherapy and exhibited high fever before being placed on a ventilator for two weeks whilst the clinicians monitored and treated her CRS (7). Despite the side effects observed, the efficacy of the treatment resulted in the CD19 CAR T cell therapy becoming the first to be approved by the United States Food and Drug Administration (FDA) for use in children and young adults in 2017 (8). As a result of CAR T cell successes, as well as an increase in the development of T cell engager (TCE) therapies in oncology and non-oncology indications, a growing interest in CRS has emerged as it represents one of the most severe adverse outcomes associated with immune-related biological therapeutics (9).

In the clinic, CRS exhibits a wide spectrum of symptoms, ranging from mild, flu-like symptoms to severe, life-threatening inflammatory responses. Milder symptoms include fatigue, headaches, fever, rash, and muscle soreness. Severe symptoms are characterized by systemic inflammation, high fever, vascular leakage, disseminated intravascular coagulation, and multi-organ system failure (1). The incidence of CRS in cancer patients receiving immunotherapy varies significantly depending on the indication and immunotherapeutic being used. Detecting and predicting CRS remains challenging, as although most patients will exhibit signs of CRS, there is no direct correlation between the severity of CRS and the therapeutic response (10). TCE therapies have varying associations with the onset of CRS, with most well-known for inducing CRS being CAR T cell and Bi-specific T cell engager (BiTE) therapies (11). CRS is also reported following immune-checkpoint inhibitor (ICI) therapies (12), however the onset of CRS in response to ICI treatments is less well known compared to other TCE therapies. Tay et al. examined clinical causes of ICI-induced CRS and interestingly found that higher grade CRS had a longer time to onset compared to patients who peaked at lower grades (1-2, using the Lee et al. scale) (12, 13). The pathology of CRS is a complex, multi-phase sequence, with distinct characteristics, suggesting a need for increased biomarker discovery and model refinement, to understand and more accurately predict the onset of CRS. This could enable identification of specific patients who may be susceptible to higher grade CRS and consequently have more severe adverse effects of immunomodulatory therapies.

**Figure 1:**
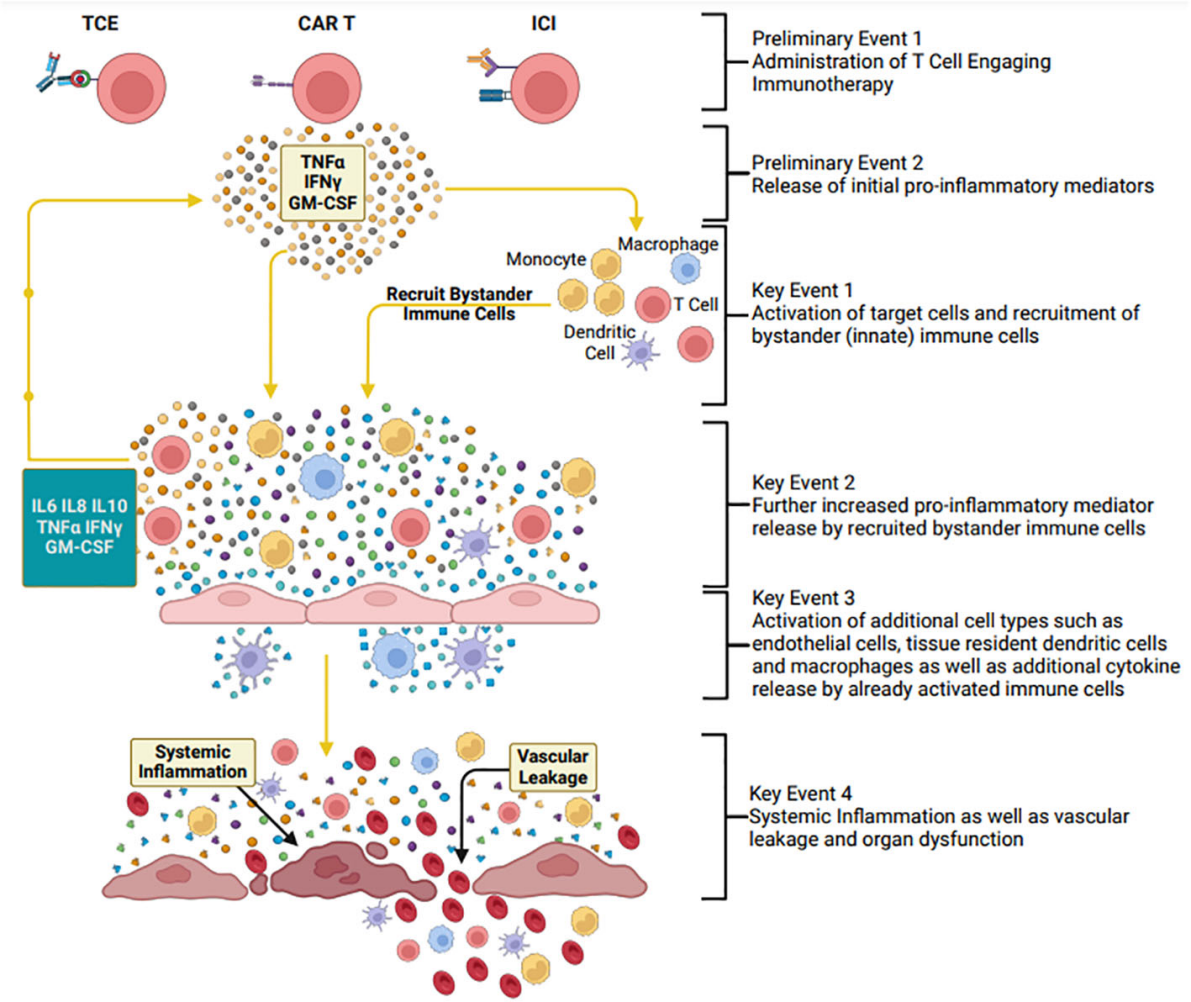
Overview of the immune-related adverse outcome pathway of cytokine release syndrome, developed by the imSAVAR consortium. Overarching depiction of the Key Events in the pathogenesis of CRS; TCE, T Cell Engager; CAR T, Chimeric Antigen Receptor T Cell; ICI, Immune-Checkpoint Inhibitor; TNFα, Tumor Necrosis Factor α; IFNγ, Interferon γ; IL, Interleukin; GM-CSF, Granulocyte-Macrophage Colony-Stimulating Factor. Created with BioRender.com.

The Immune Safety Avatar (imSAVAR) consortium is a collaboration of academic, industry, and regulatory organizations, with the aim of improving immune safety assessments of novel therapeutics. Key objectives within imSAVAR include the development and refinement of immunotoxicological models that can better predict adverse clinical outcomes, as well as the discovery of new biomarkers to aid in the prediction and management of adverse outcomes associated with biological therapies (14). The consortium highlighted CRS as a key area of focus, developing immune-related adverse outcome pathways (irAOPs) for drug-induced adverse effects. The irAOPs outline Key Events (KE) of CRS, identifying gaps that research could target to better predict and manage the onset of CRS (**Figure 1**). By identifying KEs directly linked to CRS, the development of predictive assays and models becomes possible, enabling researchers to better understand the progression of CRS and to select appropriate models for studying this complex condition. Such approaches must extend beyond mere risk assessment, aiming to provide translational relevance to predict clinical outcomes more accurately.

In 2023, the imSAVAR consortium convened to discuss the development of a comprehensive library of currently adopted CRA models, detailing the key events and models that could be used to investigate CRS in the future (**Tables 1**, **2**). Whilst recently published FDA guidance requires no further refinement of current CRS models for hazard identification, it recognizes the potential of more advanced assays such as endothelial co-cultures (48). The scope of imSAVAR is to refine current and develop new *in vitro* assays to understand the mechanisms in more depth, discover potentially relevant biomarkers and provide first-in-human (FIH) predictions. Careful model selection is important for determining the FIH dose and, depending on the context of use, of the biological therapeutic, particular models may be more suited than others.

**Table 1:** Traditional *in vitro* models for studying individual or multiple events of CRS.

Test system (Model)	Assay format	Endpoints	Key events	Selection of references
Whole blood Modified Chandler Loop CRA	2D culture in blood loops	Cytokines	MIE/1/2I	(15)
T cell + antigen stimulation (peptide pool or ‘peptivators’)	2D plate-based culture	Proliferation (e.g. CFSE)	MIE/1	(16, 17)
		Activation (e.g. CD25/CD69)	MIE/1	(16, 18)
		Cytokines	MIE/1/2	(19)
Whole blood CRA (soluble)	2D plate-based culture	Cytokines	MIE/1/2	(20–22)
Whole blood CRA (solid phase)	2D plate-based culture	Cytokines	MIE/1/2	(20–22)
PBMC CRA (soluble)	2D plate-based culture	Cytokines	MIE/1/2	(20, 23)
		Proliferation (e.g. CFSE)	MIE/1/2	(23)
		Activation	MIE/1/2	(21, 23, 24)
		Killing	MIE/1	(25)
PBMC CRA (solid phase)	2D plate-based culture	Cytokines	MIE/1/2	(20)
		Proliferation (e.g. CFSE)	MIE/1/2	(22)
		Activation	MIE/1/2	(21, 24, 26)
PBMC CRA (high density/RESTORE)	2D plate-based culture	Cytokines	MIE/1/2	(20)
		Proliferation (e.g. CFSE)	MIE/1/2	(21)
		Activation	MIE/1/2	(24, 27)
PBMC: HUVEC CRA	2D plate-based culture	Cytokines	MIE/1/2	(23)
		Soluble endothelial markers	3/4	(19, 28)
		Immune cell Activation	MIE/1	(19)
		Immune cell proliferation	MIE/1	(23)

**Table 2:** Several advanced *in vitro* models for studying individual or multiple events of CRS.

Test system (Model)	Assay format	Endpoints	Key events	Selection of references
CAR-T/Tumor cell co-cultures	2D plate-based culture	Killing	MIE/1	(29)
		Proliferation (e.g. CFSE)	MIE/1	(29)
		Activation markers (e.g. CD25/CD69)	MIE/1	(30)
		Cytokines	MIE/1/2	(29)
T cell (or PBMC)/Tumor cell co-cultures	2D plate-based culture	Killing	MIE/1	(31)
		Proliferation (e.g. CFSE)	MIE/1	(31)
		Activation markers (e.g. CD25/CD69)	MIE/1	(32)
		Cytokines	MIE/1/2	(31)
BOEC CRA	2D plate-based culture; 3D culture	Cytokines	MIE 1/2	(33)
		Vascular Leakage (3D)	4	(34, 35)
		Endothelial cell activation (3D)	3/4	(34, 35)
		Immune cell Activation (3D)	MIE/1/2	(34)
MIMIC CRA	3D culture	Cytokines	MIE/1/2	(36–38)
		Immune cell Activation	MIE/1	(36, 39–42)
MIMETAS	3D Culture	Cytokines	MIE/1/2	Not yet published - Labcorp
		Endothelial cell activation (3D)	3/4	
		Vascular Leakage (3D)	4	
		Immune cell Activation	MIE/1/2	Ongoing NC3Rs CRACK IT Challenge (43)
Draper	3D Culture	Cytokines	MIE/1/2	(44, 45)
		Endothelial cell activation (3D)	3/4	(46)
Emulate	3D Culture	Cytokines	MIE/1/2	(47)
		Endothelial cell activation (3D)	3/4	(47)
		Immune cell Activation	MIE/1/2	(47)

Current methodologies and strategies frequently include the use of *in vivo* systems to bridge standard 2D assays and clinical application (29). Although *in vivo* systems, such as humanized or genetically modified mouse models, can provide mechanistic insights, the complexity of the immune system and the variability in immune responses between species can increase the risk of therapeutic complications in the clinic (49–51). In recent years, humanized *in vitro* technologies have significantly advanced in sensitivity and physiological relevance (52), providing increased predictivity through a variety of models, tailored to the specific research questions being asked during investigative immunological safety testing. Here, we highlight a library of *in vitro* test systems that are suitable for the risk assessment of drug-induced CRS, to better understand and support the path towards clinical trials, whilst discussing the future of *in vitro* systems in drug development processes. In this review, guidance on model selection will be provided to aid future research groups based on their context of use.

## Historical evolution of traditional models of cytokine release assay

Cytokine Release Assay (CRA) has developed over several decades, since the development of techniques such as radioimmunoassay (RIA) and enzyme-linked immunosorbent assay (ELISA) (53). In the late 1990s the term “cytokine storm” began to be used to describe the excessive release of cytokines following the stimulation of immune cells (54). Cytokine storm, or Cytokine Release Syndrome (CRS) is the key driver of adverse outcomes associated with T cell Engaging therapies (55, 56).

Following the failure to predict CRS as a severe adverse event induced in Phase I clinical trials by the anti-CD28 monoclonal antibody, TGN1412 (19), several iterations of traditionally used methods and protocols for the detection of cytokine release have been developed to de-risk biotherapeutics. Stebbings et al. investigated a series of traditionally used protocols to determine the most relevant one for this compound (19). These have now been suggested as being appropriate for immunological safety assessment of a broader range of immunotherapies by the FDA, due to their more accurate recapitulation of the human responses (48). Protocol 1 (aqueous TGN1412) and protocol 2 (aqueous TGN1412 in combination with an anti-Fc antibody) conditions used PBMCs (peripheral blood mononuclear cells) and were similar to those used in the original testing for TGN1412 and failed to elicit a cytokine response. Protocol 3 also used PBMCs but involved the wet coating of TGN1412 to the base of a 96-well plate and subsequently failed to detect elevated cytokine release. Following the lack of stimulation in the initial protocols, protocols 4–6 were devised in the hope of presenting TGN1412 to the PBMCs in a manner more reflective of the *in vivo* setting and hence provide an effective stimulatory response. Both protocols 4 and 5 involved dry coating the 96-well plates, with the prior dry coating TGN1412 and the latter dry coating anti-Fc before wet coating TGN1412. Interestingly the air-dried coating (protocol 4) demonstrated a cytokine response in human whole blood, but not cynomolgus macaque whole blood. Finally, protocol 6 included an aqueous solution of TGN1412 in the presence of an endothelial cell (EC) monolayer derived from humans. Commonly, a cell line such as HUVECs (Human Umbilical Vein Endothelial Cells) is used, due to the relative ease of access and culture. Protocol 6 was designed in an attempt to mimic the effect of the interaction exhibited in the original clinical trial study patients. In this experiment, a cytokine response (TNFα and IL-8) was observed after adding TGN1412 to a co-culture of human PBMCs and endothelial cells (19). Separate work in 2011 also noted that a modified version of the aqueous assay of Protocol 1 can be used to detect the cytokine response induced by TGN1412 (27). In this modification, PBMCs are first cultured at a higher density prior to plating with aqueous compound, generating a “primed” state of T cells, akin to that generated in the lymph node, enabling an amplified cytokine reaction to TGN1412 in line with the clinical response.

Further work found the failure to predict CRS in humans in the original nonclinical work was due to the lack of CD28 expression in CD4^+^ effector memory T cells of the cynomolgus macaque species (57) used (*Macaca fascicularis*) (19, 58). Therefore, effector T cells were not stimulated in this animal model and CRS was not observed. This highlights the need for due diligence to identify pharmacologically relevant species and take phylogenetic variation in animal models into consideration for nonclinical safety assessment strategies. Additionally, it shows the limitation of animal models in predicting the safety of immune-modulatory therapeutics and the need for alternative models (14, 59) that allow for targeted design considering mechanism of action and utilizing human immune cells. For example, while assays that use only whole blood or PBMCs may well be suitable to identify the CRS risk associated with T cell engaging therapies whose target is expressed on circulating immune cells (e.g. CD19) many new therapies are in development targeting solid tumor antigens where the target is not expressed in peripheral blood. Therefore, modifications to the assay systems are absolutely required to accurately model the risk. Pharmaceutical industries and regulatory authorities have since reconsidered the requirements of safety in FIH Phase I clinical trials, with the FDA placing a greater emphasis on the utility of validated, new (animal-free) approach methodologies (NAMs) in their nonclinical evaluation of the immunotoxic potential of pharmaceuticals guidance (48).

Endothelial cells are key to the pathogenesis of CRS, releasing IL-6, a cytokine central to the progression of CRS. In addition, ECs can contribute to antibody presentation via FcyR interactions, leading to cross-linking reactions, which may contribute to specific mechanisms of CRS (60). Since TGN1412, institutions have focused on assay development to improve the physiological relevance of immunological safety testing. Two-dimensional (2D) cell culture models, as seen in Protocol 6 described by Stebbings et al. (19) are now a commonly used platform, playing a pivotal role in the development of therapeutic compounds (19). Whilst widely used for other scientific areas, TGN1412 highlighted the importance of 2D cell co-culture for immunological safety testing, leading to endothelial cells co-cultured with PBMCs or Whole Blood. The most common of these endothelial cell co-cultures for investigating CRS utilize HUVECs and iPSCs (induced Pluripotent Stem Cells) as their endothelial cell source. Although able to contribute to cytokine release *in vitro*, the heterologous nature of HUVECs can introduce HLA mismatch and generate an allogeneic immune response. The result of this is increased levels of cytokines in negative control sample groups, thus reducing the sensitivity of the overall assay (33). While full physiological relevance may not be feasible, nor required to achieve accurate clinical predictions, in the field of safety testing assay sensitivity could be important to detect incremental changes to cytokine expression, which may be indicators of CRS. The endothelial models, whilst considered more traditional models (**Table 1**), show significantly greater specificity compared to other traditional models but also lack more sophisticated model elements, such as tumor co-culture or fully autologous models.

An additional endothelial cell source, Blood Outgrowth Endothelial Cells (BOECs) are generated from PBMCs and in the presence of various growth factors and stimulants, which induce the differentiation of progenitor stem cells such that, after several weeks, BOEC colonies begin to form (33, 61). By co-culturing BOECs with PBMCs from the same donor, the Mitchell group developed a novel autologous CRA for the evaluation of known CRS-inducing compounds, ranging from mild (Campath, alemtuzumab) to severe (TGN1412) (33). This model demonstrated increased assay sensitivity compared to the more commonly used heterologous format, owing to the removal of the allogeneic immune response. This increased assay sensitivity enables the detection of more subtle cytokine changes and allows models to be used for the identification of biomarkers leading to the progression of CRS. Labcorp Drug Development, a partner within the imSAVAR consortium, have used BOECs for cytokine release assessments, and have now developed a three-dimensional (3D) vessel on chip version of the assay. The model has been established using whole blood or PBMCs in both 2D and 3D cultures, offering flexibility in model design, to suit the screening of compounds and the desired study objectives (data not yet published). The more physiologically relevant 3D design of the vessel on chip system also allows for the implementation of more complex assay endpoints, leading to further, more in-depth immunological safety testing within the scope of imSAVAR.

A modified PBMC model has been implemented by the company Immuneed (15, 62) that aims to better recapitulate the *in vivo* situation by using a modified Chandler loop protocol, which incorporates flow conditions into a whole blood assay. Briefly, whole blood is placed in plastic tubing that contains a low level of free/diffused anticoagulant (by using heparin fixed to the inner tube surface). Upon test compound addition, these tubes are then rotated in a water bath to mimic physiological flow rates and temperatures, to assess the effects of the test compounds on either coagulation or antibody interactions. By using plastic tubing that are coated with a heparin conjugate, the need for high concentrations of anticoagulants can be avoided in this system, which allows for the contribution of both the complement and coagulation pathways to immune cell activation to be assessed. Using tool monoclonal antibodies, which are known to induce CRS in patients (e.g. OKT3, ANC28.1, Adalimumab), a lower background and consequently increased sensitivity of the cytokine responses were noted when compared to standard plate-based assays. However, as the assay platform also lacks an endothelial cell component the important contribution of these cells cannot be assessed.

The implementation of flow and additional tissues, such as vasculature, is of great physiological importance, particularly in CRS, given that the Key Events (KEs) involve the activation of endothelial and immune cells. The recruitment of additional immune cells is an important element during CRS progression. Static MPS models, such as 3D tissues from MatTek™, have recapitulated many biological functions, such as cytotoxicity and oxidative stress responses, whilst modelling several disease states (63, 64). These 3D models present powerful methods for exploring developmental controls at a molecular level for drug development, as well as the pathogenesis of diseases. The sophistication of 3D tissue models has provided the basis for regulatory changes towards using alternative models where animal models can be avoided (65). 3D models alone, however, do not recapitulate true human physiology, as they may lack several key attributes, including certain tissue-tissue interactions, the circulation of immune cells, vasculature, interstitial flow, and organ-specific attributes. These are crucial for accurately mimicking systemic measures such as absorption, distribution, metabolism, and excretion (ADME) and pharmacokinetics and pharmacodynamics (PK/PD). Static 3D MPS therefore may not always be optimal for the accurate assessment of drug disposition, efficacy and toxicity within the human body, but can provide valuable data in other applications (66). To support 3D static models, microfluidic 3D systems and organ-on-chip (OOC) technologies are able to enhance the physiological relevance by implementing flow, allowing some of these applications to be included, such as vasculature, which plays an important role in CRS. The OOC platforms provide further physiological relevance, however single organ platforms are still unable to provide all the aforementioned requirements to study CRS fully. Advanced OOC systems, for example those with additional components such as vasculature or multi-organ functionalities, are beginning to address systemic applications that were previously only possible in *in vivo* animal studies and the clinic (67–69).Whilst traditional assays for the assessment of immunological safety testing have been around for several decades, the refinement of specific detailing within these assays has advanced in more recent years, such as the autologous nature of the BOEC CRA assay. This highlights the relevant role that traditional models for CRA assessment still have within the development of immune-engaging therapeutics, and their potential use cases to support/provide useful alternatives in place of unavailable New Approach Methodologies (NAMs) depending on the study context of use and compound mechanism of action.

## Advanced *in vitro* systems and microphysiological approaches

The increased interest in the development of advanced *in vitro* systems has prompted a belief amongst research groups that the reduction of animal models is possible, supported by the FDA’s recently updated legislation to encourage the use of *in vitro* alternatives, where possible, over animal models (70, 71). Advanced *in vitro* models come in various “packages”, whether it be multi-organ on a chip systems or highly sensitive platforms, using a blend of 2D and 3D models to suit the relevant application. The most well-known OOC platforms attempt to recapitulate human microphysiology and allow for more predictive therapeutic screening of compounds, which is gradually becoming a more relevant focus for immunological safety testing groups (65). The majority of these “packages” could be adapted to investigate individual key events of CRS (**Table 2**), for example running time course assays with a flow cytometry component to detect the endothelial cell activation in KE3.

Sanofi, a member of the imSAVAR consortium, developed the Modular Immune *In Vitro* Construct (MIMIC^®^) which is an *in vitro* system that enables testing of the immune response to vaccines, biologics or any product likely to interact with the immune system directly in a human-based system. It comprises a series of culture assays that can be used to assess both human innate and adaptive immunity (37, 39–41). For the analysis of intravenously (IV) delivered products, Sanofi developed the MIMIC CRA, which is an automated 3D co-culture assay system consisting of fresh red blood cell (RBC)-depleted whole blood cells, autologous plasma, and a human endothelial cell line (EA.hy926) that is pre-cultured to confluency on a collagen bed (currently type I bovine collagen) (42). The system has been employed in the past to retrospectively evaluate the cytokine profiles induced by TGN1412 (38), various T cell engager (TCE) molecules under development (72, 73) and recently for CAR T cell therapy (42). The MIMIC CRA platform possesses relevant attributes that differentiate it from the more traditional platforms employed for similar types of evaluation. Utilizing fresh RBC depleted whole blood cells enhances the diversity of cell types available in the system. Using autologous plasma along with serum-free media for treatments makes certain important human serum components, like complement, available in the system during testing. Indeed, this system offers greater fidelity and sensitivity for evaluating cytokine activity of biologics (presumably through the contribution of plasma components, granulocytes, and/or other immune factors) (74). Additionally, endothelial cells serve as a target organ by being an important source of IL-6, one of the major pro-inflammatory cytokines, hence amplifying the inflammatory response (33). The major readout for the MIMIC CRA platform is the cytokine/chemokine secretion profile. As a secondary readout, flow cytometry based phenotypic pre and/or post analysis can also be performed on fresh RBC depleted whole blood cells, as well as cells harvested from MIMIC^®^ CRA cultures after 20–22 hours of incubation with biologics. This makes the Sanofi MIMIC^®^ system applicable to studying the majority of the key events outlined in the irAOP for CRS, with minor adaptations required.

MIMETAS is a 3D microfluidic developer with several platforms relating to immunological research, suitable for CRAs. Their products support vessel on chip capabilities, comprising of single or multiple channels to seed and grow vessels alongside an extracellular matrix compartment. Organoids can also be supported through the OrganoPlate^®^ GRAFT platform, enabling the vascularization of the organoids to support more physiologically relevant therapeutic delivery (75). This range of systems aids the development of specific, advanced *in vitro* models, suitable for studying CRS and other immune-related areas of research. This is demonstrated through collaborations through the CRACK-It challenge, with other industry partners and the NC3Rs (National Centre for the Replacement, Refinement & Reduction of Animals in Research) seeking to develop more reproducible OOC models (43). The close physiological relevance that OOC developers have achieved supports the 3Rs (Reduction, Replacement and Refinement of animals in research) and demonstrates increased abilities to monitor and detect the onset of CRS (76, 77). The OrganoPlate^®^ 2-lane 96 MIMETAS platform is the current system used by Labcorp Drug Development, in the previously discussed 3D BOEC model for assessing CRS, with collaborations across the imSAVAR consortium using other 3D models to investigate biomarkers for CRS after immunotherapy dosing. These collaborations are comparing (not yet published) several differences between traditional cell models and advanced *in vitro* models, such as the effects of flow implementation and 2D versus 3D model structures. It is important to note that MIMETAS are not the only 3D microfluidic platform to implement vasculature into their models (68, 78).

Using the Draper PREDICT96 system, Gard et al. (44) demonstrated a 28-day model for investigating gingival tissue inflammatory responses and recovery (44). The PREDICT96 system uses integrated pump mechanisms to circulate assay fluids through respective channels at highly customizable flow rate/patterns, whilst also supporting a high throughput. Gard et al. monitored key markers involved in gingival inflammation, many of which overlap with CRS, such as TNFα, IFNγ, GM-CSF, IL-6, IL-8 and IL-10. This demonstrates the ability to use this system to measure cytokine release in a variety of different tissues by substituting the endothelial cells and tissue type used, as seen in a variety of other work from the PREDICT96 platform (45, 46, 79). Since the covid-19 pandemic, the PREDICT96 platform has also demonstrated promising data for studying viral infections, being the first documented human tissue lung on a chip for studying SARS-CoV-2 infection and viral replication. COVID-19 is of interest with regards to CRS as the inflammatory response in both conditions is comparable (45). CRS can occur as a result of severe COVID-19 infection following similar pathology to CRS induced by T cell engaging immunotherapies (80, 81). The diversity in the Draper PREDICT96 platform therefore allows for a vast variety of applications that could be exploited for in-depth cytokine release assessment and biomarker discovery to better understand and manage the on-target toxicity often associated with immunotherapies.

Emulate is another OOC company with ground-breaking models for various tissues and disease states. The Emulate lung model was the first OOC system to accurately replicate complex organ-level physiological and pathophysiological responses (82). More recently, Goyal et al. (47) used an Emulate chip to replicate the formation of lymphoid follicles, which form during chronic inflammation and immune responses. They demonstrated that their chip could evaluate the efficacy of seasonal vaccines and monitor clinically relevant cytokine biomarkers released in response to multiple antigens (47).

Advancements in assay technologies and 3D cell culture techniques have allowed research to thrive in the biotechnology field, leading to the emergence of multiple, advanced models for immunological safety testing and CRS. The complexity of the immune system is becoming more understood through several collaborative groups, such as imSAVAR, working to develop models capable of predicting clinical outcomes. Animal models have traditionally been used in immunological testing; however, the emergence of advanced *in vitro* MPS models could begin to reduce or replace animal models in the future as regulatory bodies are beginning to see the benefit and favor the enhanced safety testing that human *in vitro* immunological models can provide.

## Delivering new approaches for cytokine release assay

The development of NAMs to enhance immunological safety testing has demonstrated impressive progress in recent years, but there are still several overarching questions regarding their suitability, such as “Are they clinically relevant enough?” or “Do they provide high enough throughputs for drug development?”. With regards to clinical relevance, NAMs are frequently discussed from the perspective that full alignment with human physiology is required. This may not be the most appropriate way to measure the suitability of NAMs, nor may it be a relevant perspective at all. NAMs approaches, such as MPS and OOC have shown enhanced clinical relevance across several toxicological fields (68, 75, 83) but not every aspect is truly relevant. For example, there are OOC platforms that are restricted to single or low-number organ models, which could limit the systemic relevance of a system compared to that of a human or animal, but not always. Whilst multiple single-organ models could be used for toxicological screening, these may miss therapeutic markers/associated risks that could be observed in systemic settings such as cumulative unexpected stimulation of immune cells leading to enhanced CRS symptoms, or toxic metabolites causing unexpected immunostimulatory effects. It should be noted that these potential complications are not solely associated with NAMs, but also the traditional models, and suggests a cautious approach when transitioning away from animal testing, as NAMs become more widely adopted with regulators. This emphasizes the need to strategically select the most appropriate models for your desired immunological screening assays. Some MPS and OOC platforms however, have produced enhanced clinical relevance and assay sensitivity without achieving full physiological recapitulation, demonstrating improved concordance with humans. There is currently large expectations on NAMs to fill a wide variety of nonclinical toxicological testing, for which there will not be one model that accomplishes all of these different qualities. However, advancements in the technologies to provide a library of enhanced assay sensitivity, biomarker predictions and physiological relevance is progress towards achieving the aims of significantly reduced adverse effects and improved safety confidence, when entering FIH trials.

To provide new immunological safety testing models, the scalability must also be considered. Industry typically requires medium to high throughput methods to enable the screening of multiple testing compounds at once. MPS and OOC platforms are generally of a low to medium throughput, depending on the model used. Whilst this is not ideal on the surface, the readout capabilities and increased assay sensitivity, compared to traditional and animal studies may result in more efficient biotherapeutic developments overall. Whilst the *in vitro* screening may be slow in throughputs, the reduced/replaced *ex vivo* and *in vivo* studies required will enable an accelerated pathway to FIH trials, not only in experimental time, but also in animal licensing and ethical approval documentation drafting. Careful consideration of rapidly updating regulatory guidelines require careful monitoring to ensure appropriate model selection to address the context of use in mind for a particular therapeutic (48, 84). For context, further advantages and disadvantages of both traditional and advanced models are shown in **Table 3**.

**Table 3:** Suggested advantages and disadvantages of previously mentioned models.

Model	Advantages	Disadvantages
Whole blood Modified Chandler Loop CRA *^1^	Includes flow, allows for physiological clotting	Has an air-fluid interface and complex flow pattern, making standardization is more difficult
T cell + antigen stimulation (peptide pool or ‘peptivators’) *^1^	Enhanced reproducibility and standardization.	Lacks some physiological relevance by bypassing natural antigen processing pathways.
Whole blood CRA (soluble) *^1^	Utilizes the whole blood fraction, providing enhanced physiological relevance and enables studies such as complement-interacting compounds	Reduced sensitivity versus PBMC assays, does not recapitulate endothelial compound presentation
Whole blood CRA (solid phase) *^1^	Utilizes the whole blood fraction and provides enhanced physiological relevance and enables studies such as complement-interacting compounds. Recapitulates endothelial compound presentation	Reduced sensitivity versus PBMC assays, does not recapitulate endothelial compound presentation
PBMC CRA (soluble) *^1^	Increased sensitivity versus whole blood assays	Does not recapitulate endothelial compound presentation
PBMC CRA (solid phase) *^1^	Increased sensitivity versus whole blood assays. Capable of recapitulating endothelial compound presentation	Relies on compound manipulation to recapitulate endothelial compound presentation
PBMC CRA (high density/RESTORE) *^1^	Increased sensitivity versus whole blood assays. Recapitulates lymph node-like activity by “priming” PBMCs	Does not recapitulate endothelial compound presentation
PBMC: HUVEC CRA *^1^	Increased sensitivity versus whole blood assays. Recapitulates endothelial compound presentation without the need for compound manipulation	Potential increased background cytokine response from graft versus host
CAR-T/Tumor cell co-cultures *^2^	Enables study of human cell therapies in human representative contexts, as opposed to modified animal models *in vivo*. enables study of compound interaction within disease context	It is difficult to recapitulate full tumor microenvironment *in vitro*. Lacks additional cells such as immune and endothelial cells. The effector to target ratios are most of the time artificially established, compared to the *in vivo* situation
T cell (or PBMC)/Tumor cell co-cultures *^2^	Enables study of compound interaction within disease context	Difficult to recapitulate full tumor microenvironment *in vitro*
BOEC CRA *^2^	Recapitulates endothelial compound presentation without the need for compound manipulation. Comprises a fully autologous system to potentially reduce graft versus host response. Enables development of disease-state models through generation of endothelial cells from disease populations	Potential for greater variability in results due to donor-specific endothelial responses (could make statistical interpretation more difficult and require larger datasets)
MIMIC CRA *^2^	Provides a fully human, high-throughput, and predictive platform for pre-clinical immune response screening	Lacks advanced cell capabilities such as organ on chip. Typically designed for vaccine screening, but can be applied to alternative measurements
MIMETAS *^2^	Incorporates fluid flow, is compatible with imaging and provides high-throughput analysis options. Range of plates to suit various applications such as vessel and organ on chip	Gravity-based fluid flow (lower sheer stress). Bi-directional fluid flow depending on specific platform used
Draper *^2^	Incorporates tuneable and unidirectional fluid flow, is compatible with imaging and provides high-throughput analysis options	Currently not available for direct purchase but partnerships with the company are possible to use the platform
Emulate *^2^	Incorporates tuneable and unidirectional fluid flow and is compatible with imaging	Requires separate incubation and flow unit and is not high throughput.

*^1^ Traditional Models.

*^2^ Advanced Models.

## Discussion

Nonclinical immunotoxicology testing has traditionally relied heavily on animal models. Regulatory guidance, such as ICH-S6 and ICH-S8, require a range of studies to ensure the safety of a novel biologic, particularly due to the complexity of the challenge to understand immune related hazards and risk associated with molecules targeting the immune system. As such there has been a growing interest in establishing comparable *in vitro* alternatives that are suitable models for the potential activation of the immune response. This is now reflected in the recent FDA CDER nonclinical guidance for immunotoxicology in industry, which details a range of different study types, including T cell proliferation, complement activation, and *in vitro* cytokine release (48). Since the late 2000’s the rise in available advanced *in vitro* models has provided an opportunity to further enhance immunological testing strategies, following optimization and validation for the desired context of use.

The composition of major phenotypes of the immune system can vary significantly between nonclinical species and humans, leading to translational challenges. This has previously led to some hazards not being identified at the nonclinical phase, resulting in undesirable clinical findings. However, alongside *in vivo* models, *in vitro* alternatives could help to minimize the risk and likelihood of clinical shortcomings in the future via a weight of evidence-based approach. It is true that in some cases animal models remain the most suitable models in predicting clinical outcomes, however the rapidly advancing *in vitro* technology discussed, coupled with the growing awareness and desire to reduce animal usage in research in favor of humanized models is beginning to reduce the reliance on animals to accurately predict human responses in the clinic. With more targeted therapies, it also has become more common that pharmacological activity is lacking in any animal species, further increasing the need for *in vitro* models to support the nonclinical safety assessment.

For the successful development of novel biologics or cell therapies, several nonclinical objectives must be considered prior to the FIH clinical trials. First of these should be the provision of sufficient evidence and data to determine a safe starting dose via the minimum anticipated biological effect level (MABEL), and where possible, highlight any potential toxicity that may need further management. Relevant data to better predict a safe starting dose is key for the success of the trial and the safety of the volunteers (patients), a vital element of which is highlighting any CRS potential a new compound may exhibit. As such, having models to better understand these biological processes is imperative. The research interests in immunological safety testing, particularly for understanding CRS *in vitro* and lymphoid related activities from organizations such as the NC3Rs CRACK IT, ILSI HESI, NHRI, BioSafe, IHI/imSAVAR and multiple MPS developers aim to provide data, using NAMs, that are thought to be more representative of clinical outcomes than current nonclinical models. Historically cytokine release risk management has been developed through primarily clinical experience; however, *in vitro* methods are now emerging. For example, the 2019 study by Sachdeva et al. who reversed CRS after CART cell treatment by blocking GM-CSF, preventing monocyte-dependent release of CRS mediators (85).

**Figure 2:**
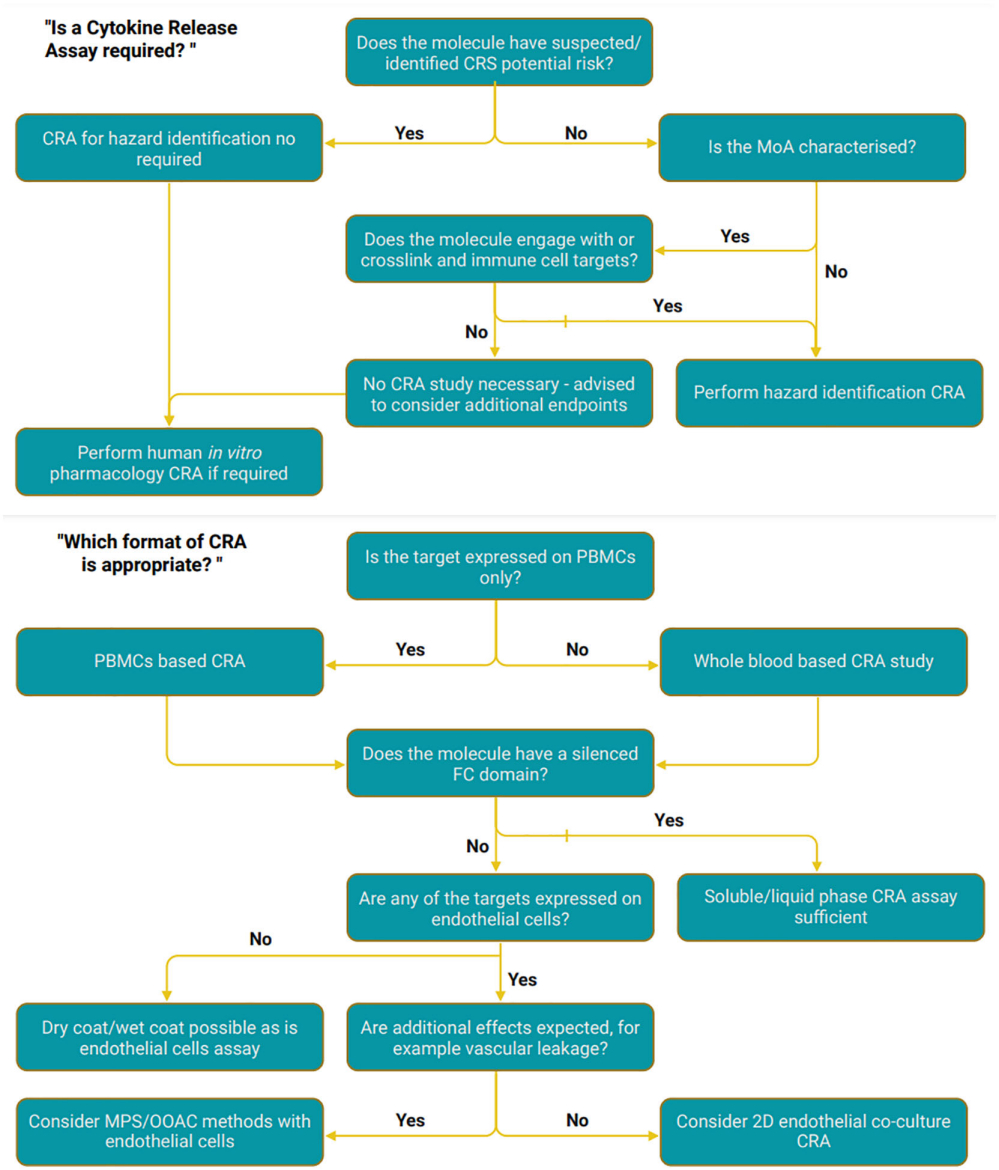
Decision tree to aid appropriate model selections. Created with BioRender.com.

The aforementioned study is an example of the universal vision that a large proportion of the immunology community shares: to develop and refine models to better inform clinical outcomes. In 2023, FDA guidelines for immunological safety testing were updated to recognize the utility of a number of different assays, including endothelial co-culture assays and the use of patient derived cells (48). This is a significant step forward, in recognizing the development of more advanced *in vitro* test systems such as co-culture, microphysiological and OOC platforms. This will add to the ever-growing library of test systems, not just for immunotoxicology but also the wider toxicological research fields, enabling more specificity with regards to model selection based off the required context of use. An example of this would be the nonclinical species-specific drug-induced liver injury recently demonstrated using the Emulate OOC platform (86). The system Jang et al. developed provided the screening of suitable species prior to *in vivo* and clinical studies, to assess the translatability from animal to human where animal models were required by the appropriate regulatory organization (86). This insight into which model offers increased translatability provides researchers with a viable *in vitro* model to help assess toxicological properties. The liver is also a key organ to be aware of with regards to CRS, due to late-stage CRS having a significant, toxic effect upon liver tissue and function (87). Therefore, the sophisticated liver model from Emulate could be adopted to assess late-stage CRS effects, further widening the context of use from a modelling perspective. Nevertheless, the selection of a suitable advanced *in vitro* model is complex, and there are several factors to consider (for example cell line selection, extracellular matrix materials, implementation of flow, level of throughput and tissue complexity) which are also dependant on the context of use. Recent guidance released by the FDA (48) has begun to address the lack of an updated guidance since the 2006 TGN1412 incident, but the ever expanding *in vitro* model library can make model selection a challenging process. To relieve some complexity regarding model choice, the imSAVAR consortium have outlined a proposed aid in the form of a decision tree (**Figure 2**), which is supported by recent literature and, highlights some of the methodologies that could be applied to *in vitro* CRS studies in the future. The decision tree is intended to guide model selection for both academic and industry organizations assessing immune cell engaging therapies.

For modalities where cytokine release is an expected part of their mechanism of action, e.g. T cell engagers, *in vitro* assays including classical cytokine release assay formats have been used to determine the MABEL dose for FIH clinical trials. However, as the cytokine release in these assays is highly influenced by the experimental set-up (e.g. effector-to-target cell ratio, assay duration etc.) the selection of the MABEL dose can be considerably lower than the pharmacologically active dose in humans. Improved *in vitro* models and selection of the appropriate model using the suggested decision tree (**Figure 2**) may allow for more accurate safe starting dose selection. Importantly, in oncology, where FIH trials are conducted with patients, a more accurate prediction of the safe starting dose may allow more patients to receive pharmacologically active doses. As these improved *in vitro* models are further developed and more widely accepted the requirement for validation by pharmaceutical industries will need to be considered. Alongside this, it would be expected that regulatory bodies further implement these animal alternative models into their guidance which would in turn support their validation with guidance from irAOPs designed specifically for animal alternative model selections.

Model selection is a complex process and there isn’t always a clearly defined path for conducting immunotoxicological studies. Therefore, narrowing potential model options down to a category of testing platforms using the decision tree (**Figure 2**), with no specific category of model preferred over another, could aid research groups. It is important to note that the model selection is also dependant on the intended context of use. Depending on whether the intention is to gain an understanding of the molecule’s mechanism of action or is instead for hazard identification, for example, the requirements of a model may vary. Further to this, the regulatory requirements and downstream use of the data must also be taken into account: is the data for information purposes only, or for critical decision making ahead of Investigational New Drug Application (IND) submission? Depending on this context, the way in which the study is conducted could differ. For example, if a CRA is required for molecule screening and candidate selection of a molecule, how this data is used varies significantly from a lead candidate hazard identification study where data will contribute to MABEL calculations prior to FIH trials. Furthermore, for a molecule where there are no relevant species and the CRA is standalone as opposed to supporting a toxicology study, regulatory bodies may require the study to be performed to Good Laboratory Practice (GLP). In immune-oncology applications, for example to assess tumor cell targeting of an immunotherapy such as CAR T cells, the inclusion of additional cell types other than the CAR T cells and tumor cells would be less of a necessity, and a more simplified and higher-throughput model, with the implementation of flow, could be more favorable. There are also several clinical areas with regards to CRS that are often currently overlooked in *in vitro* and *in vivo* testing. For example, the tumor burden, which influences cytokine release, as well as the lymphodepletion patients often go through prior to CAR T therapy could be factored into some of the models discussed here. Simplified hazard identification assay models may not be sufficient to cover the full extent and onset of CRS, and recent guidance advises considering the mechanism of action of a molecule to appropriately select the most biologically relevant assay format to recapitulate the full response (48). In this regard, as it is known that endothelial cells contribute to the totality of the CRS response, an assay format incorporating this cell type would be highly advantageous. In summary, the decision tree shown in **Figure 2**, is designed to aid model selection in an expanding *in vitro* library of testing platforms, highlighting where more traditional methodologies may be sufficient rather than advanced microphysiological systems that require specialist training or equipment.

The functionality of certain NAMs has demonstrated great potential for improving the predictability of human responses, with regards to CRS. Whilst there has been significant improvement in more accurate clinical predictions, it remains important to select an appropriate model based around the mechanism of action and the key events of CRS likely to be encountered, to accurately understand the specific effects of a biological therapy. As NAMs become more readily available and adopted, updates to any guidance on the appropriate model selection will inevitably be required. This review of models for CRS serves as an early guide to aid researchers’ decision-making for immunological safety testing with regards to their context of use.

## Future directions

Immunological safety testing has seen rapid advancements in recent years, providing greater concordance with human responses. The changes in regulatory (65, 70) and governmental (84) guidance in favor of NAMs as alternatives to animals (where appropriate) and their eventual replacement in certain scientific fields highlights the significant progress that advanced *in vitro* models have made. Following the FDA, other regulators are expected to update their guidance on the phasing out (or reduction) of animal models in research in the near future, placing a larger emphasis on NAMs with enhanced clinical relevance. This will require further amendments to the context of use proposed here, to accommodate NAMs model library expansions for immunological safety testing, and potential incorporation of patient derived cells to support personalized medicine approaches. The current ability of NAMs to provide increased hazard identification sensitivity, as well as complex mechanistic understandings of human responses to immune cell engaging therapies, has formed a strong foundation for future model development. With continued advances, confidence when entering FIH trials will be greatly enhanced through stronger clinical predictability, solidifying the incorporation of NAMs in drug development pipelines.
